# LT-IIb(T13I), a Non-Toxic Type II Heat-Labile Enterotoxin, Augments the Capacity of a Ricin Toxin Subunit Vaccine to Evoke Neutralizing Antibodies and Protective Immunity

**DOI:** 10.1371/journal.pone.0069678

**Published:** 2013-08-02

**Authors:** Christopher J. Greene, Chrystal M. Chadwick, Lorrie M. Mandell, John C. Hu, Joanne M. O’Hara, Robert N. Brey, Nicholas J. Mantis, Terry D. Connell

**Affiliations:** 1 The Witebsky Center for Microbial Pathogenesis and Immunology, The University at Buffalo, Buffalo, New York, United States of America; 2 The Department of Microbiology and Immunology, The University at Buffalo, Buffalo, New York, United States of America; 3 Division of Infectious Disease, Wadsworth Center, New York State Department of Health, Albany, New York, United States of America; 4 The Department of Biomedical Sciences, University at Albany, Albany, New York, United States of America; 5 Soligenix, Inc., Princeton, New Jersey, United States of America; Federal University of São Paulo, Brazil

## Abstract

Currently, there is a shortage of adjuvants that can be employed with protein subunit vaccines to enhance protection against biological threats. LT-IIb(T13I) is an engineered nontoxic derivative of LT-IIb, a member of the type II subfamily of heat labile enterotoxins expressed by *Escherichia coli,* that possesses potent mucosal adjuvant properties. In this study we evaluated the capacity of LT-IIb(T13I) to augment the potency of RiVax, a recombinant ricin toxin A subunit vaccine, when co-administered to mice via the intradermal (i.d.) and intranasal (i.n.) routes. We report that co-administration of RiVax with LT-IIb(T13I) by the i.d. route enhanced the levels of RiVax-specific serum IgG antibodies (Ab) and elevated the ratio of ricin-neutralizing to non-neutralizing Ab, as compared to RiVax alone. Protection against a lethal ricin challenge was also augmented by LT-IIb(T13I). While local inflammatory responses elicited by LT-IIb(T13I) were comparable to those elicited by aluminum salts (Imject®), LT-IIb(T13I) was more effective than aluminum salts at augmenting production of RiVax-specific serum IgG. Finally, i.n. administration of RiVax with LT-IIb(T13I) also increased levels of RiVax-specific serum and mucosal Ab and enhanced protection against ricin challenge. Collectively, these data highlight the potential of LT-IIb(T13I) as an effective next-generation i.d., or possibly i.n. adjuvant for enhancing the immunogenicity of subunit vaccines for biodefense.

## Introduction

Due to the recent rise in incidents of bioterrorism in the United States and across the globe, there is an urgent need to develop safe and effective vaccines against a range of biological threat agents. One of the major concerns is ricin, a potent, bipartite toxin derived from the castor bean plant [*Ricinus communis*]. Ricin toxin is easily isolated from castor beans using simple enrichment steps and is extremely toxic to humans whether injected, inhaled, or ingested [1], [2]. Due to its potent toxicity and ease of dissemination, the Centers for Disease Control and Prevention (CDC) has designated ricin to be a Category B toxin.

Ricin is a member of the type II ribosome inactivating protein family of toxins. The ricin toxin A subunit (RTA) is an RNA N-glycosidase that depurinates a conserved adenosine residue located within the sarcin/ricin loop (SRL) of eukaryotic 28s ribosomal RNA (rRNA) [1]. Depurination of this residue results in an immediate cessation of ribosome progression, which subsequently inhibits protein synthesis. The ricin toxin B subunit (RTB) binds with micromolar affinity to α(1–3)-linked galactose and N-acetylgalactosamine residues that are expressed on the surface of all mammalian cell types. Binding of RTB to these receptors mediates internalization and retrograde transport of the ricin holotoxin to the endoplasmic reticulum (ER). In the ER, RTA dissociates from RTB and is retrotranslocated across the ER membrane into the cytosol where it gains access to rRNA targets [2], [3]. In addition to ribosome inactivating properties, ricin also elicits vascular leak syndrome (VLS), which primarily afflicts endothelial cells [4], [5].

Although a considerable amount of effort has been expended on developing post-exposure treatments for ricin intoxication, immunization against ricin remains the most reasonable and reliable method to ensure long-lasting protection for military forces, first responders, and research personnel [6]–[11]. Past and current efforts to develop a safe, effective vaccine include ricin toxoid [12], deglycoslated RTA [13], and a truncated form of RTA known as RV*Ec* [14]. One of the most promising candidate ricin vaccines currently in development is RiVax [15], [16], a non-toxic, recombinant derivative of RTA in which tyrosine 80 (Y80A) and valine 76 (V76M) were mutated to eliminate RTA’s RNA N-glycosidase and VLS activities, respectively. Studies in mice and rabbits demonstrated that RiVax is safe and immunogenic when administered by the intramuscular (i.m.) and intradermal (i.d.) routes [16]–[19]. In a Phase I clinical trial, however, not all participants responded to RiVax when employed as an i.m. vaccine [20]. In a second Phase I trial in which individuals received three i.m. immunizations over a span of 26 weeks, it was demonstrated that adsorption of RiVax to aluminum salts (“alum”) adjuvant enhanced RiVax-specific serum antibodies (Ab) titers. Yet, the levels of anti-RiVax Ab were neither robust nor long lasting, especially in the low dose group (10 µg) [21]. Moreover, levels of toxin-neutralizing Ab were also extremely low in RiVax-immunized individuals, which is problematic considering that these Ab are likely the primary determinant of protective immunity to ricin. Collectively, these data underscored the need to identify new adjuvants to boost the efficacy of RiVax.

Some of the most potent adjuvants described to date belong to the families of bacterial heat-labile enterotoxins (HLT). The type I and type II HLT expressed by enterotoxigenic *Escherichia coli* (LT, LT-IIa, LT-IIb, and LT-IIc) and by *Vibrio cholerae* [cholera toxin, (CT)] are composed of a single, enzymatically active A subunit that is non-covalently bound to a pentameric array of B subunits [22]. The A subunit of HLT is a potent ADP-ribosylase that targets the G_sα_ regulatory protein of the adenylate cyclase system. The B pentamer of HLT mediates binding of the holotoxin to ganglioside receptors, which are a family of cell surface glycolipids found ubiquitously on mammalian cells. Each HLT binds to a unique ganglioside or with varying affinity to members of a subset of gangliosides. For example, CT and LT bind avidly to ganglioside GM1 [23]. In contrast, LT-IIb binds with highest affinity to GD1a and less to GM2 and GM3 [24].

A number of studies revealed that the type II HLT LT-IIb is a potent mucosal and systemic adjuvant. Intranasal (i.n.) immunization of mice with model antigens (Ag) in combination with LT-IIb induces robust Ag-specific immune responses at local mucosal sites, distal mucosal sites, and systemically [25]–[28]. The enthusiasm for the potential use of LT-IIb and other HLT as i.n. adjuvants has been reduced, however, by concerns of the inherent toxicity of these molecules. For that reason, considerable effort has been expended toward developing safe and effective HLT mutants that lack toxicity, yet retain the potent adjuvant properties of the native proteins. Substitution of the threonine at amino acid position 13 in the B polypeptide of LT-IIb with an isoleucine [LT-IIb(T13I)] dramatically reduced the binding affinity of that mutant HLT to its respective ganglioside receptors and dramatically reduced the toxicity of the HLT to levels that were undetectable by standard bioassays [26], [29], [30]. The reduced binding affinity for ganglioside receptors did not ablate the capacity of LT-IIb(T13I) to bind to various immune cells, including macrophages, CD8^+^ T cells, CD4^+^ T cells, and B cells. It is not surprising, therefore, that when used as an i.n. adjuvant, LT-IIb(T13I) exhibited immunomodulatory properties that were similar to those observed for native LT-IIb [26].

Trafficking and cellular studies have shown that some HLT, when administered by the i.n. route, have the propensity to traffic to the brain via the olfactory nerve, which has been correlated with an increased risk of facial nerve pathologies, ostensibly due to elicitation of neuroinflammation [31]. As a result of potential trafficking and neuroinflammation, an impetus to evaluate alternative routes of administration of HLT adjuvants has evolved. Skin is a site of high immune surveillance and Ag sampling, two functions that are crucial for eliciting robust systemic immune responses [32]–[34]. Thus, increased focus has been applied to the skin as a potential route for inducing immune responses to foreign Ag. Initial investigations revealed that i.d. administration of LT-IIa, a member of the type II HLT subfamily, and of LT-I, a member of the type I HLT subfamily, had the capacity to enhance Ag-specific immune responses against ovalbumin, a model Ag. Notably, however, immunization using each of those HLT induced significant amounts of inflammation at the administration site, a side-effect that is unacceptable for an i.d. immunization strategy [35].

The goal of this study, therefore, was to investigate the capacity of LT-IIb(T13I) to enhance the immunogenicity of RiVax. The data suggest that LT-IIb(T13I) has exploitable properties as an effective, low-inflammatory i.d. adjuvant for enhancing protective immune responses against ricin and other biothreat agents.

## Materials and Methods

### Chemicals and Reagents

Recombinant His-tagged LT-IIb and LT-IIb(T13I) were purified using previously described methods [26]. All preparations were determined to be essentially free of lipopolysaccaride (<0.03 ng/µg of protein; *Limulus* amoebocyte assay kit, Charles River Endosafe, Charleston, SC). Ricin was purchased from Vector Laboratories (Burlingame, CA). RiVax™ was provided by Dr. Robert Brey (Soligenix Inc., Princeton, NJ). Rat anti-mouse CD45 primary Ab was purchased from BD Pharmingen (San Diego, CA). Rabbit anti-rat collagen type I Ab was purchased from Chemicon International Inc. (Temecula, CA). Chicken anti-rat Alexa647 and chicken anti-rabbit Alexa488 Ab, DAPI, and SlowFade Gold antifade reagent (S36936) were purchased from Invitrogen (Grand Island, NY). Imject®, an alum adjuvant, was purchased from Pierce (Rockford, IL). Protease inhibitor cocktail was purchased from Research Products International (Prospect, IL).

### Choice of Experimental Animal

Mice were chosen as subjects for investigation of immune responses by HLTs since (a) mice constitute a well-established model for immunological studies, (b) there is considerable background information on their immune systems, (c) a wide range of high-quality immunochemical and cellular reagents is available, and (d) a number of potentially useful mutants (genetic knock outs and transgenics) are available. The smallest number of animals were employed, consistent with the numbers shown in the past to yield statistically valid data.

### Veterinary Care

All mice used in this study were housed under conventional, specific pathogen-free conditions and were treated in strict compliance with guidelines established by the Institutional Animal Care and Use Committees (IACUC) at the University at Buffalo and at Wadsworth Center, New York State Department of Health. Experimental animal facilities at The University at Buffalo and at Wadsworth Center are managed by full-time professional veterinarians who may be called upon for advice and assistance. Both facilities are fully accredited by the American Association for the Accreditation of Laboratory Animal Care (AALAC), and comply with NIH policy, the Animal Welfare Act, and all other applicable federal, state, and local laws.

### IACUC Approval and Procedures

All procedures to be used have been approved by the local IACUC at The University at Buffalo and the Wadsworth Center. Mice were housed and maintained according to the prescribed standards, under the supervision of the IACUC at The University at Buffalo and the Wadsworth Center.

### Euthanasia

Mice were euthanized by exposure to carbon dioxide asphyxiation and cervical dislocation.

### Immunizations

Eight to twelve week old, female BALB/c mice were purchased from Harlan Laboratories (Madison, WI) or Taconic Laboratories (Hudson, NY). For i.d. immunizations, groups of 5 mice were anesthetized with 75 mg/kg of ketamine and 10 mg/kg of xylazine or isoflurane, fur was removed on their dorsum, and the underlying skin was cleaned using alcohol swabs. Using insulin syringes, mice were intradermally immunized with PBS (vehicle control), RiVax (0.5 or 5.0 µg), or various combinations of RiVax with 1.0 µg of LT-IIb, 1.0 µg of LT-IIb(T13I), or adsorbed to Imject® in 10 or 50 µL volumes for low (0.5 µg) and high (5.0 µg) doses of RiVax, respectively. For i.n. immunizations, a well-established mouse mucosal immunization model was employed [25], [26], [28], [30]. Groups of 5 unanesthetized mice were immunized by the i.n. route with PBS (vehicle control), RiVax (0.5 or 5.0 µg), or RiVax in combination with 1.0 µg of LT-IIb or LT-IIb(T13I). Immunizations were administered in standardized volumes that were applied to both external nostrils (5 µl/naris) with mice being rested for 5 minutes between each nasal administration. The i.n. and i.d. immunization regimens consisted of a primary immunization followed by booster immunizations administered at day 10 and day 20.

### Collection of Serum and Secretion Samples

Blood collected from the tail vein, submandibular vein, or by cardiac puncture at the time of euthanization was centrifuged at 4°C for 20 minutes at 16,000 RCF, after which serum fractions were collected and stored at −80°C. Saliva samples were collected with a micropipetter after stimulation of salivary flow by injecting each mouse intraperitoneally with 5.0 µg of carbachol (Sigma-Aldrich Co., St. Louis, MO). Mice were placed into individual cages and fecal samples were collected and frozen at −80°C until needed. To prepare fecal material for Ab analysis, 100 mg of fecal pellets were dissolved in 400 µL PBS containing protease inhibitors. After extensive vortexing, samples were centrifuged at 16,000 RCF for 5 min at RT. Supernatant was collected for analysis. Samples of lung lavages were collected at the time of euthanization. Briefly, mice were anesthetized with isoflurane and exsanguinated by cardiac puncture. Pulmonary circulation was cleared by flushing 3 mL of PBS through the ventricles until the lungs visibly blanched. Blunt ended syringes were inserted into the trachea and tied off using sutures. Lungs of each animal were flushed with 1 mL of PBS. Bronchio-alveolar lavage (BAL) fluid was collected in microfuge tubes and frozen at −80°C.

### RiVax and RTA Ab Analysis

Levels of isotype and subclass anti-RiVax Ab in serum, saliva, lung lavage, and feces were measured by ELISA. Nunc Immulon 2HB polystyrene 96-well microtiter plates (ThermoFisher Scientific) coated with 100 µL RiVax (5 µg/mL) per well were incubated overnight at RT. To determine total IgA concentrations, plates were coated with 100 µL unlabeled goat anti-mouse IgA specific Ab (1 mg/mL) (Southern Biotechnology Assoc., Birmingham, AL). After blocking with PBS containing 0.15% Tween-20 and 1% bovine serum albumin (Amresco, Solon, OH), serial two**-**fold dilutions of serum or secretion samples were added in duplicate and plates were incubated overnight at RT. Plates were washed with PBS-Tween and incubated at RT for 4 h with the appropriate alkaline phosphatase-conjugated goat anti-mouse Ig isotype or subclass-specific Ab (Southern Biotechnology). Plates were washed and developed with nitrophenyl phosphate substrate (Amresco) and the reaction was terminated by the addition of 100 µl/well of 2N NaOH. ELISA plates were read on a VersaMax microplate reader at 405 nm wavelength and analyzed with SoftMax Pro 5.4 (Molecular Devices, Sunnydale, CA). Concentrations of Ag-specific and total IgA Ab were calculated by interpolation of calibration curves generated using a mouse Ig reference serum (ICN Biomedicals, Aurora, IL). Salivary IgA responses are reported as the percentage of RiVax-specific IgA in total IgA to compensate for variation in salivary flow rate.

Anti-RTA ELISAs were performed using previously established methods [36]. Briefly, wells of Nunc Maxisorb F96 microtiter plates (ThermoFisher Scientific) were coated overnight with 100 µL RTA (1 µg/mL) in PBS (pH 7.4) prior to being treated with sera from the immunized mice. Horseradish peroxidase-labeled goat anti-mouse IgG-specific polyclonal Ab (Southern Biotechnology) was employed as a secondary detection reagent. ELISA plates were developed using the colorimetric detection substrate 3,3′,5,5′-tetramethylbenzidine (Kirkegaard & Perry Labs, Gaithersburg, MD) and were analyzed using a SpectroMax 250 spectrophotometer and Softmax Pro 5.2 software (Molecular Devices).

### Detection of Ricin-neutralizing Ab

Vero cell cytotoxicity assays were performed using a standard assay [37]. Briefly, Vero cells were trypsinized, adjusted to approximately 5×10^4^ cells per ml, and seeded (100 µl/well) into white bottom 96-well plates (Corning Life Sciences, Corning, NY), and allowed to adhere overnight. Vero cells were treated with ricin (10 ng/ml), ricin:serum mixtures, or with culture medium (negative control) for 2 h at 37°C. Cells were washed to remove non-internalized ricin or ricin:serum mixtures and were incubated for 48 h. Cell viability was assessed using CellTiter-GLO reagent following manufacturer’s protocol (Promega, Madison, WI). All treatments were performed in triplicate and 100% viability was defined as the average value obtained from wells in which cells were treated with culture medium only. Neutralizing data is presented as the reciprocal serum dilution required to protect 50% of ricin treated cells.

### Ricin Challenge

Groups of mice were immunized on days 0, 10, and 20 (and 34 for i.n.) by the i.d. or i.n. route with 0.5 µg of RiVax in the presence or absence of 1.0 µg of LT-IIb(T13I). On day 27 (i.d.) and 41 (i.n.), serum samples were analyzed for anti-RTA and ricin-neutralizing Ab. One week later, mice were challenged with 10 LD_50_ (100 µg/kg) of ricin by intraperitoneal (i.p.) injection and monitored for 96 h for survival. As a surrogate marker of ricin intoxication, blood glucose measurements were obtained just prior to ricin challenge and then every 24 h thereafter for 72 h. Blood samples were obtained from the tail vein and blood glucose levels were measured using an Aviva ACCU-CHEK handheld blood glucometer (Roche, Indianapolis, IN). Mice that became overtly moribund or when blood glucose levels fell below 25 mg/dl were euthanized. For statistical purposes, readings below the meter’s limit of detection of 12 mg/dl were assigned that value.

### Analysis of Skin Inflammation

Mice immunized by the i.d. route with RiVax and adjuvants were observed every 24 h for reactivity at the immunization site. For gross morphologic analysis, indurations resulting from i.d. immunization were measured daily for 14 days using digital calipers. Skin sections were collected for histological analysis from separate groups of mice at a time point 7 days after i.d. immunization. Skin sections were fixed overnight in 10% buffered formalin, embedded in paraffin, and sliced into sections of 5 µm thickness. Unstained sections were employed for subsequent immunofluorescence analysis. Other sections were stained with hematoxylin and eosin (H&E) for light microscopic analysis. For light micrographs of H&E stained sections, images were acquired using a Nikon Eclipse E600 Epifluorescence microscope at 40x and 200x magnification. Unstained sections were processed for immunostaining [38]. Briefly, Ag retrieval of sections was performed by treating the sections for 20 min at 95°C in 10 mM citrate buffer (pH 6). After cooling to RT, sections were washed in PBS and blocked with 2% (W/V) powdered nonfat milk for 1 h at RT, followed by additional washes in PBS. Sections were incubated overnight at 4°C with dilutions (1∶100) of primary rat anti-mouse CD45 and rabbit anti-mouse collagen 1 Ab in 1% (W/V) powdered nonfat milk. After washing in PBS, sections were incubated for 1 h at RT with 1∶500 dilutions of chicken anti-rat Alexa647 and chicken anti-rabbit Alexa488 secondary Ab (Invitrogen). After washing in PBS, sections were mounted onto glass slides using SlowFade Gold mounting medium containing DAPI nuclear stain (Invitrogen) and analyzed using a Zeiss Axioimager fluorescence microscope at 200x magnification. Fifteen random images were obtained throughout each section and the number of CD45^+^ immune cells and the total number of nuclei were counted using Axiovision and ImageJ software.

### Statistical Analysis

Data were statistically analyzed using Excel 2008 (Microsoft, Redmond, CA) and Prism 5 (GraphPad Software, Inc., San Diego, CA). Unpaired Student’s t-tests were performed to analyze differences between two groups and survival curves were analyzed using the Logrank test. Analysis of variance and Bonferroni’s multiple-comparison test were used for comparisons across multiple groups.

## Results

### I.d. Co-administration of LT-IIb or LT-IIb(T13I) with RiVax Enhances RiVax-specific Ab and Ricin-neutralizing Ab

**Figure 1:**
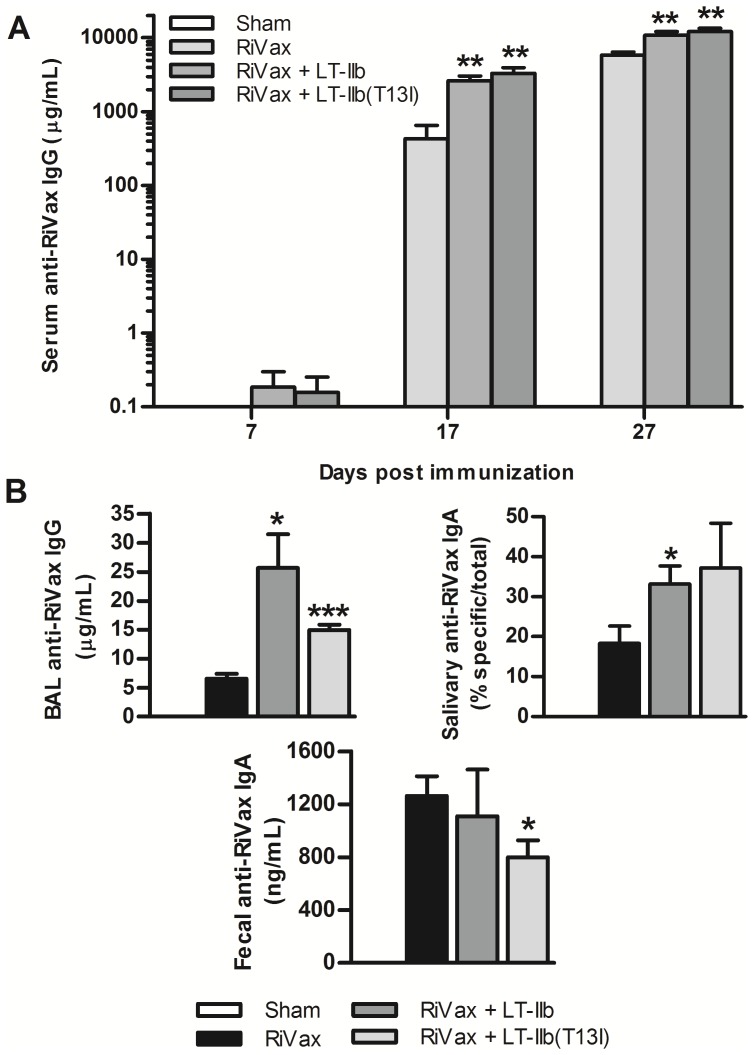
LT-IIb and LT-IIb(T13I) enhance the immune response to RiVax when co-administered by the i.d. route. Mice were immunized intradermally with 5.0 µg of RiVax alone or with 1.0 µg of LT-IIb or LT-IIb(T13I) on days 0, 10, and 20. (A) Level of Ag-specific IgG Abs in sera from immunized mice on days 7, 17, and 27. (B) Level of RiVax-specific IgG and IgA Abs obtained on day 27 in BAL, salivary, and fecal samples from immunized mice. Data (n = 5) presented as the arithmetic mean with error bars denoting one S.E.M on a logarithmic scale (Y-axis). Key: *, p<0.05; **, p<0.01; ***, p<0.001 compared to RiVax alone. Data were compared using an unpaired Students t-test.

To evaluate the capacity of LT-IIb and LT-IIb(T13I) to enhance Ag-specific immune responses when administered by the i.d. route, mice were immunized on days 0, 10, and 20 with RiVax in the presence or absence of either adjuvant. RiVax alone was moderately immunogenic when administered by the i.d. route, as evidenced by the fact that measurable levels of anti-RiVax serum IgG were detected on days 17 and 27. The addition of LT-IIb or LT-IIb(T13I) elicited a seven to eight-fold increase in anti-RiVax IgG Ab at day 17 and a two-fold increase at day 27 (**Fig. 1A**), demonstrating that both the wt and detoxified LT-IIb mutant had i.d. adjuvant properties. Additionally, mice immunized with RiVax in combination with either LT-IIb exhibited higher levels of anti-RiVax IgG in BAL fluid and elevated levels of RiVax-specific IgA in saliva, as compared to mice immunized with RiVax alone (**Fig. 1B**). These data demonstrated that LT-IIb and LT-IIb(T13I) are potent adjuvants for RiVax when administered by the i.d. route.

**Figure 2:**
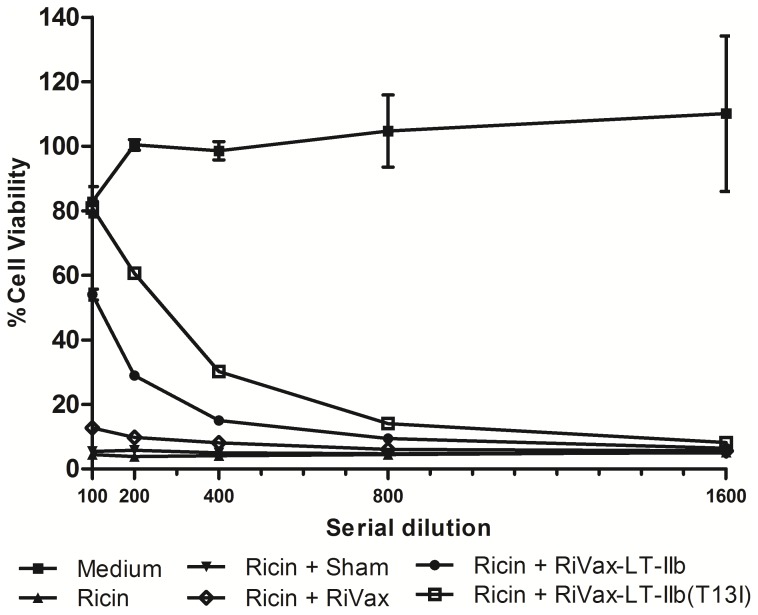
I.d. Immunization of RiVax with LT-IIb or LT-IIb(T13I) enhances ricin-neutralizing Ab production. Sera from immunized mice were assessed for the capacity to neutralize ricin in a Vero cell cytotoxicity assay. Ricin (10 ng/mL) was incubated with serum for 30 min and the mixture was applied in triplicate to Vero cells grown in 96-well microtiter plates for 2 h at 37°C. After washing, fresh media was applied and cell viability was assessed 48 h later. Data shown is representative for each group of immunized animals (n = 5) with the error bars denoting one S.E.M. of the average of three replicate wells of treated Vero cells.

Since it is well established that in mice ricin-neutralizing Ab are the primary determinant of protective immunity to ricin, an *in vitro* cytotoxicity assay [37] was employed to determine if LT-IIb or LT-IIb(T13I) enhanced the production toxin-neutralizing activity (TNA) when co-administered with RiVax. Mice immunized with 5.0 µg of RiVax in the absence of adjuvant exhibited no detectable levels of TNA in sera, despite notable levels of total RTA-specific Ab (**Table 1**; **Fig. 2**). This observation was not completely unexpected since **(i)** neutralizing Ab constitute only a small fraction of the total Ag-specific Ab elicited by immunization with RiVax and **(ii)** the in vitro assay used to assess TNA is relatively insensitive [37]. In contrast, TNA was detected in the sera of 80% of mice that had been immunized with 5.0 µg of RiVax in combination with LT-IIb or with LT-IIb(T13I), although the RTA-specific titers were comparable to those observed in mice immunized solely with RiVax. These data demonstrated that co-administration of 5.0 µg of RiVax with LT-IIb or detoxified LT-IIb(T13I) with RiVax qualitatively and quantitatively enhances ricin-specific Ab responses.

**Table 1:** Ricin-neutralizing Ab titers on day 27 after i.d. immunization with high and low doses of RiVax.

High dose (5.0 µg)			Low dose (0.5 µg)		
Group and mouse #	RTA titers^a^	TNA^b^	Group and mouse #	RTA titers^a^	TNA
Sham	0	0	RiVax #1	0	0
RiVax #1	64000	0	RiVax #2	1000	0
RiVax #2	64000	0	RiVax #3	8000	0
RiVax #3	32000	0	RiVax #4	0	0
RiVax #4	128000	0	RiVax #5	0	0
RiVax #5	64000	0	RiVax+LT-IIb(T13I) #1	16000	0
RiVax+LT-IIb #1	128000	50	RiVax+LT-IIb(T13I) #2	16000	25
RiVax+LT-IIb #2	64000	0	RiVax+LT-IIb(T13I) #3	16000	0
RiVax+LT-IIb #3	64000	100	RiVax+LT-IIb(T13I) #4	8000	50
RiVax+LT-IIb #4	64000	50	RiVax+LT-IIb(T13I) #5	32000	25
RiVax+LT-IIb #5	128000	100	Control #1	0	0
RiVax+LT-IIb(T13I) #1	128000	50	Control #2	0	0
RiVax+LT-IIb(T13I) #2	128000	800	Control #3	0	0
RiVax+LT-IIb(T13I) #3	64000	50	Control #4	0	0
RiVax+LT-IIb(T13I) #4	128000	200	Control #5	0	0
RiVax+LT-IIb(T13I) #5	64000	0			

a Values are reciprocal end point titers, as described in Materials and Methods.

b TNA were determined in a Vero cell cytotoxicity assay, as described in Materials and Methods.

### LT-IIb(T13I) Enhances Protective Immunity to Ricin when Co-administered Intradermally with RiVax

Based on the capacity of LT-IIb and LT-IIb(T13I) to augment serum TNA when co-administered by the i.d. route with RiVax, we hypothesized that the adjuvants would enhance immunity to ricin challenge even when animals were immunized with a dose-sparing amount of RiVax. To evaluate that hypothesis, mice were primed on day 0 and then boosted on days 10 and 20 with a low dose of RiVax (0.5 µg), alone or in combination with LT-IIb(T13I) (1.0 µg). LT-IIb(T13I) was evaluated in this model and not LT-IIb, as the former adjuvant proved as effective as the latter adjuvant at stimulating TNA in the prior immunization experiments.

**Figure 3:**
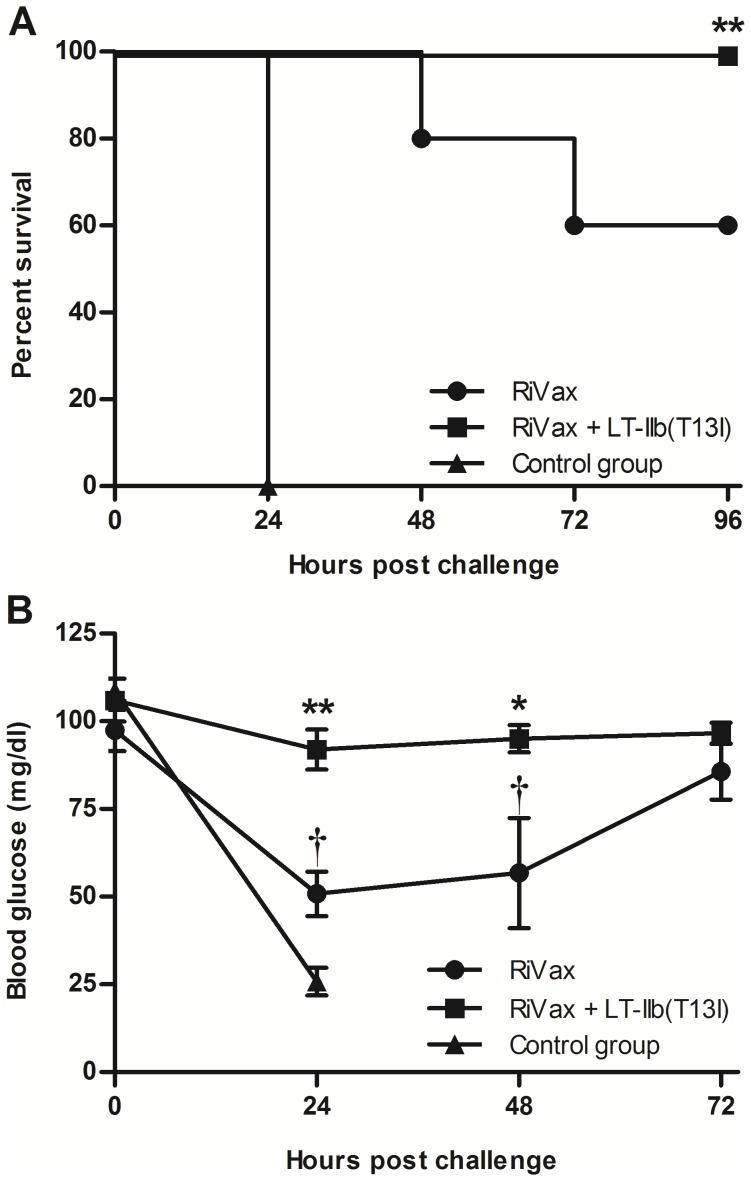
Administration of LT-IIb(T13I) by the i.d. route enhances protective immunity to ricin. Mice were immunized intradermally on days 0, 10, and 20 with 0.5 µg of RiVax in the presence or absence of 1.0 µg of LT-IIb(T13I) and challenged 14 days after the final immunization with 10 LD_50_ of ricin. (A) Survival of immunized mice after i.p. challenge with ricin. Data were compared by the Logrank test. (B) Blood glucose levels of immunized mice during challenge with ricin. Data presented as the arithmetic mean with error bars denoting one S.E.M. (n = 5). Key: *, p<0.05; **, p<0.01 compared to RiVax; †**,** p<0.05 compared to 0 h. Data were compared using an unpaired Student’s t-test.

In this low dose immunization scheme, only two of the five mice immunized solely with RiVax seroconverted; none of the mice had detectable levels of serum TNA (**Table 1**). In contrast, all mice immunized with RiVax and LT-IIb(T13I) produced high titers of anti-RTA serum Ab and three of the five produced serum TNA (**Table 1**). To assess protective immunity elicited by these immunization regimens, mice were challenged two weeks after the final immunization with 10 LD_50_ of ricin by i.p. injection using a well-established challenge regimen [37]. Within 24 h, all of the sham-immunized mice succumbed to ricin intoxication. By 72 h, 40% of mice that been immunized solely with RiVax had died of ricin intoxication, whereas all of the RiVax-LT-IIb(T13I) immunized mice survived (**Fig. 3A**). Moreover, mice that were co-administered RiVax and LT-IIb(T13I) experienced no statistical reduction in blood glucose levels following exposure to ricin. This is in contrast to the surviving RiVax-immunized animals, which experienced significant drops in blood glucose levels at 24 and 48 h after challenge (**Fig. 3B**). These data established that LT-IIb(T13I) has the capacity to significantly enhance the protective ability of RiVax when administered intradermally.

As a side note, several of the mice immunized solely with RiVax survived ricin challenge, despite any detectable TNA in their sera prior to challenge. We and others have observed this phenomenon (*i.e.*, immunity in the absence of detectable serum neutralizing antibodies) previously and have attributed to the relative insensitivity of the Vero cell-based cytotoxicity assay used to measure TNA. We cannot, however, formally rule out the possibility that alternative (innate or adaptive) mechanisms of ricin toxin neutralization are operating *in vivo* that are not reflected in standard *in vitro* Vero cell-based TNA assays (e.g., Fc-mediated clearance).

### LT-IIb(T13I) is Superior to Inject®

Alum has been used for decades as a strong intramuscular adjuvant. To compare the relative strength of LT-IIb(T13I) to alum, groups of mice were immunized i.d. with RiVax (0.5 µg) in the presence of LT-IIb, LT-IIb(T13I), or Imject®, a commercially available adjuvant consisting of aluminum hydroxide and magnesium hydroxide. LT-IIb was included in these studies to assess the degree to which detoxification of LT-IIb(T13I) impacted immunogenicity and local inflammation.

**Figure 4:**
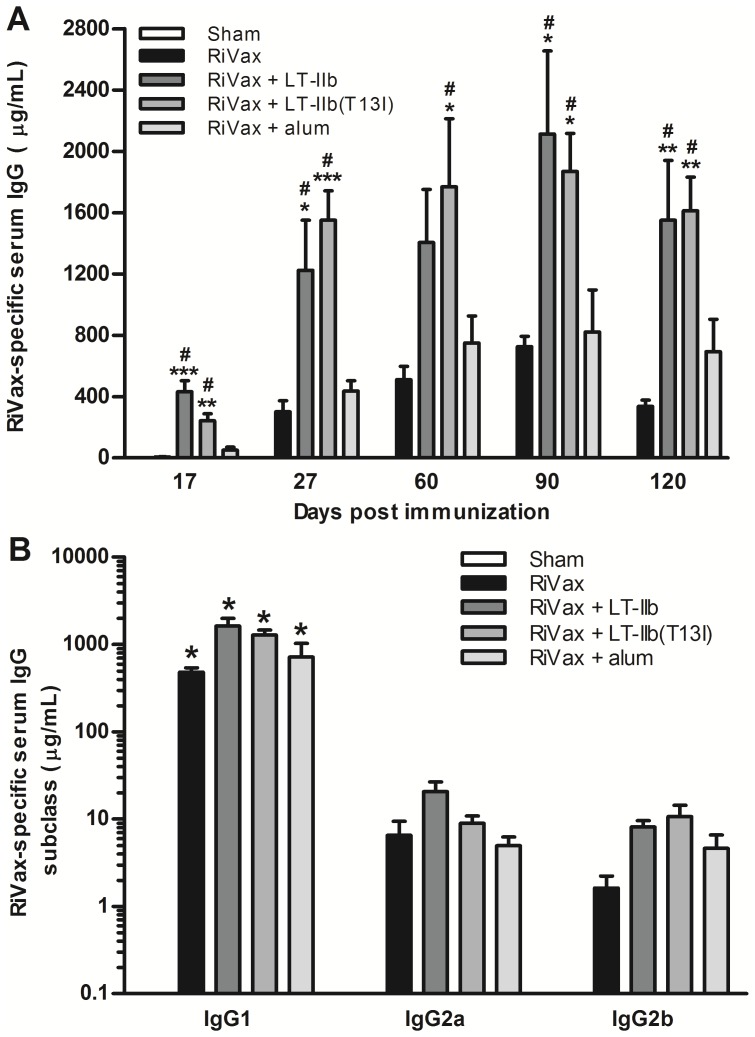
LT-IIb(T13I) is superior to alum (Imject®) at enhancing immune responses to RiVax when administered intradermally. Mice were immunized intradermally on days 0, 10, and 20 with 0.5 µg of RiVax, RiVax adsorbed to Imject®, or RiVax combined with 1.0 µg of either LT-IIb or LT-IIb(T13I). (A) Level of RiVax-specific serum IgG Ab from days 17, 27, 60, 90, and 120. (B) RiVax-specific IgG subclass analysis from the sera of immunized mice taken on day 90. Data are presented as the arithmetic mean with error bars denoting one S.E.M. (n = 5). Key: (A) *, p<0.05; **, p<0.01; ***, p<0.001 compared to RiVax; #, p<0.05 compared to RiVax+alum. (B) *, p<0.001 compared to IgG2a and IgG2b from the same group. Data were compared using ANOVA.

Mice that received RiVax in combination with LT-IIb(T13I) demonstrated significantly higher levels of anti-RiVax serum IgG Ab at all time points examined in comparison to mice that received only RiVax or RiVax in combination with Imject® (**Fig. 4A**). Moreover, LT-IIb(T13I) was no less effective than LT-IIb in enhancing anti-RiVax Ab. All immunization regimens skewed RiVax-specific Ab towards the IgG1 subclass (**Fig. 4B**). Collectively, these data indicated that LT-IIb(T13I) is superior to Inject® at stimulating RiVax-specific Ab when co-administered by the i.d. route.

### Local Inflammation Following Immunization with LT-IIb(T13I)

**Figure 5:**
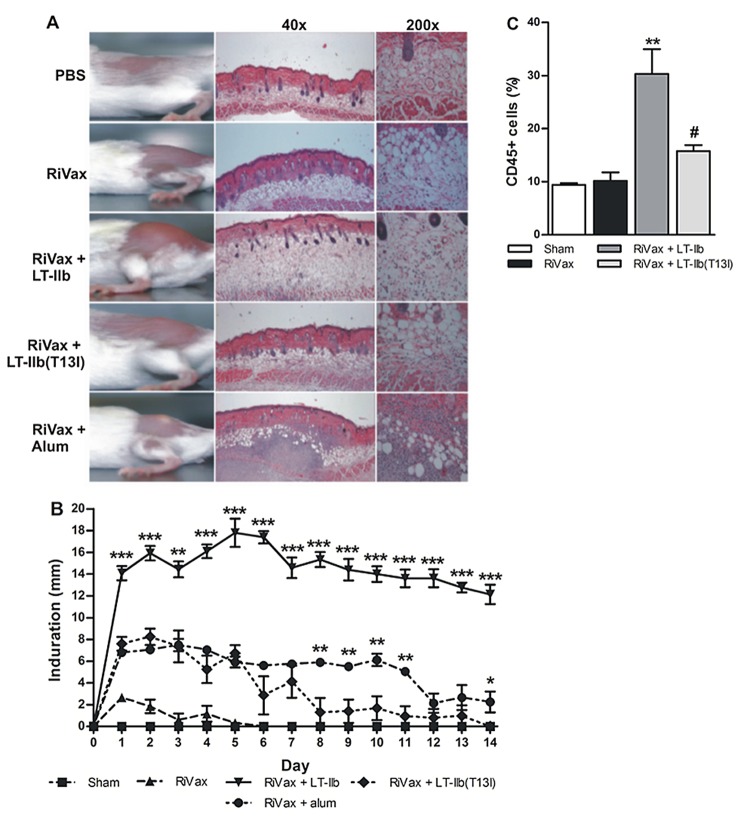
LT-IIb(T13I) is minimally inflammatory in comparison to LT-IIb. Mice were immunized by the i.d. route with 5.0 µg of RiVax, RiVax adsorbed to Imject®(alum), or RiVax combined with 1.0 µg of either LT-IIb or LT-IIb(T13I). (A) Gross morphologic photos and H&E stained micrographs of typical inflammation at the site of injection on day 7 post-immunization (n = 5). (B) Level of inflammatory induration at the injection site (n = 5). (C) Percent of CD45^+^ immune cells normalized to the total number of cells counted using Axiovision and ImageJ software from 15 random fluorescent micrographs per skin section (n = 3). Data are presented as the arithmetic mean with error bars denoting one S.E.M. Key: significance in comparison to LT-IIb(T13I) *, p<0.05; **, p<0.01; ***, p<0.001. Data in B were compared using an unpaired Student’s t-test; data in C were compared using ANOVA; #, p<0.05 - significance in comparison to LT-IIb; **, p<0.01 - significance in comparison to RiVax.

To assess the degree to which LT-IIb(T13I) induces inflammation following i.d. injection, we examined the extent of inflammatory responses at the injection sites over the course of two weeks. RiVax induced a very low-inflammatory response, as evidenced by minor indurations in the skin that dissipated after a few days (**Fig. 5**). At 7 days post-immunization, mice administered RiVax in combination with LT-IIb had large indurations at sites of injection that were accompanied by extensive fluid accumulation and cellular infiltration, particularly in the adipose layer of the skin (**Figs. 5A and 5B**). In contrast, only minimal inflammatory responses were associated with injection of LT-IIb(T13I). Microscopic analysis of skin sections taken 7 days post-immunization confirmed that LT-IIb(T13I) was much less inflammatory than LT-IIb. In fact, the histology sections from mice injected with RiVax-LT-IIb(T13I) were comparable to the sections from mice injected solely with RiVax (**Fig. 5A**). Substantial amounts of cellular infiltration with minimal edema were observed in mice that received RiVax in combination with Imject®.

Not surprisingly, LT-IIb and LT-IIb(T13I) also exhibited differences in the duration of the inflammatory responses. Whereas inflammation associated with administration of LT-IIb persisted beyond 14 days, LT-IIb(T13I)-associated inflammation had fully subsided by day 8. In fact, during the first 7 days post-immunization, the extent of inflammation elicited by LT-IIb(T13I) was similar to that elicited by Imject®. By the second week, however, LT-IIb(T13I)-associated inflammation was greatly reduced, whereas the indurations induced by Imject® persisted (**Fig. 5B**).

As a further means to quantify the extent of inflammation, skin samples at the sites of immunization were analyzed by immunofluorescence staining. An enhanced number of CD45^+^ immune cells were observed at the injection sites of mice co-administered RiVax and LT-IIb (30% of total cells) in comparison to the numbers of cells found in the sites that had received only RiVax or RiVax in combination with LT-IIb(T13I) (**Fig. 5C**). Additionally, the accumulation of immune cells induced by LT-IIb(T13I) was significantly less than the accumulation of cells induced by LT-IIb. The excessive amount of cellular infiltration and autofluorescence induced by Imject® abrogated the ability to accurately quantify the numbers of immune cells in the skin of those mice. Collectively, these data established that LT-IIb(T13I), a potent i.d. adjuvant, has minimal inflammatory properties.

### LT-IIb(T13I) enhances the immunogenicity and protective ability of RiVax when co-administered by the i.n. route

**Figure 6:**
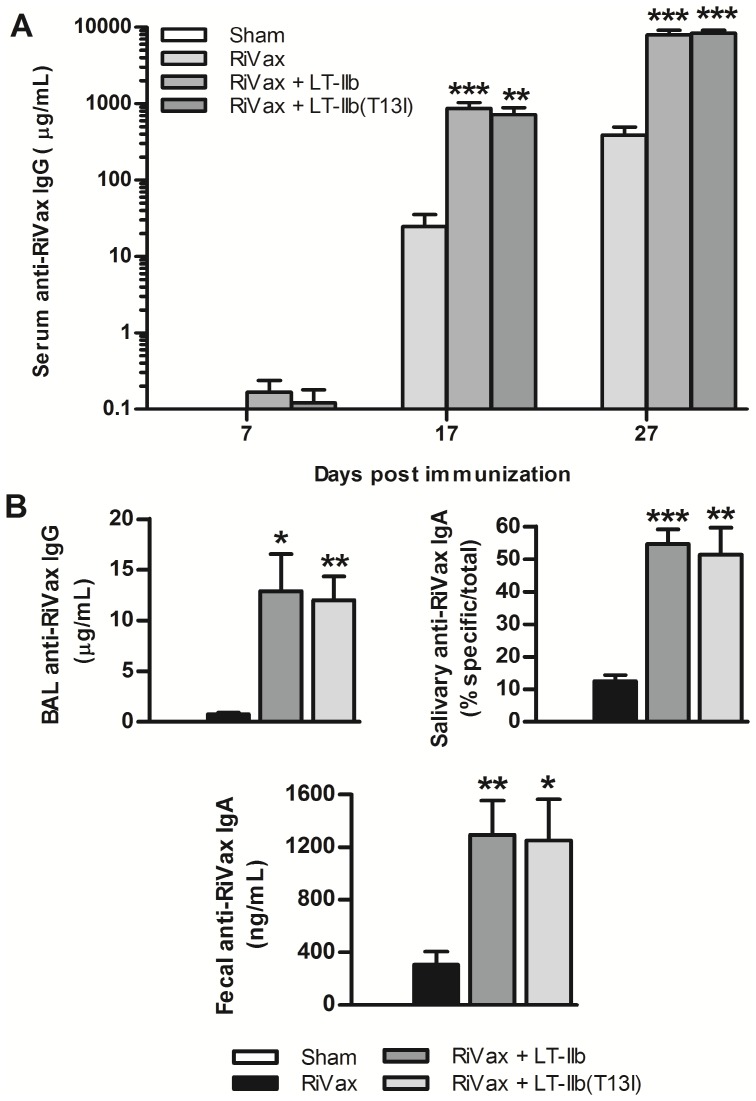
Immunization with LT-IIb(T13I) by the i.n. route enhances RiVax-specific Ab production. Mice were immunized intranasally with 5.0 µg of RiVax alone or with 1.0 µg of LT-IIb or LT-IIb(T13I) on days 0, 10, and 20. (A) Level of RiVax-specific IgG Ab in sera from immunized mice on days 7, 17, and 27. (B) Level of RiVax-specific IgG and IgA Ab obtained on day 27 from lung lavage, saliva, and fecal samples from immunized mice. Data (n = 5) are presented as the arithmetic mean with error bars denoting one S.E.M on a logarithmic scale (Y-axis). Key: *, p<0.05; **, p<0.01; ***, p<0.001 compared to RiVax. Data were compared using an unpaired Student’s t-test.

The innate toxicity of LT-IIb precludes its usefulness as an i.n. adjuvant. In contrast, LT-IIb(T13I), due to its lack of detectible toxicity, may enable this mutant molecule to be employed as a safe and effective i.n. adjuvant. To determine if LT-IIb(T13I) augments Ab responses against RiVax when administered by a mucosal route, mice were intranasally immunized with 5.0 µg of RiVax in the presence or absence of LT-IIb(T13I). LT-IIb was included in these studies as a control adjuvant. I.n. immunization with RiVax elicited very low levels of Ag-specific Ab. In contrast, i.n. co-administration of RiVax with LT-IIb(T13I) significantly elevated RiVax-specific Ab levels above those levels observed in mice that were intranasally immunized only with RiVax as early as 1 week after the booster immunization (day 17) (**Fig. 6A**). In addition, LT-IIb(T13I) enhanced the anti-RiVax IgG and IgA Ab responses in BAL fluid, saliva, and fecal samples, each of which represent anatomical sites that are susceptible to ricin exposure (**Fig. 6B**). Interestingly, LT-IIb(T13I) was no less effective as an adjuvant than LT-IIb, thereby underscoring that the T13I mutation in LT-IIb effectively uncouples toxicity and adjuvanticity.

**Figure 7:**
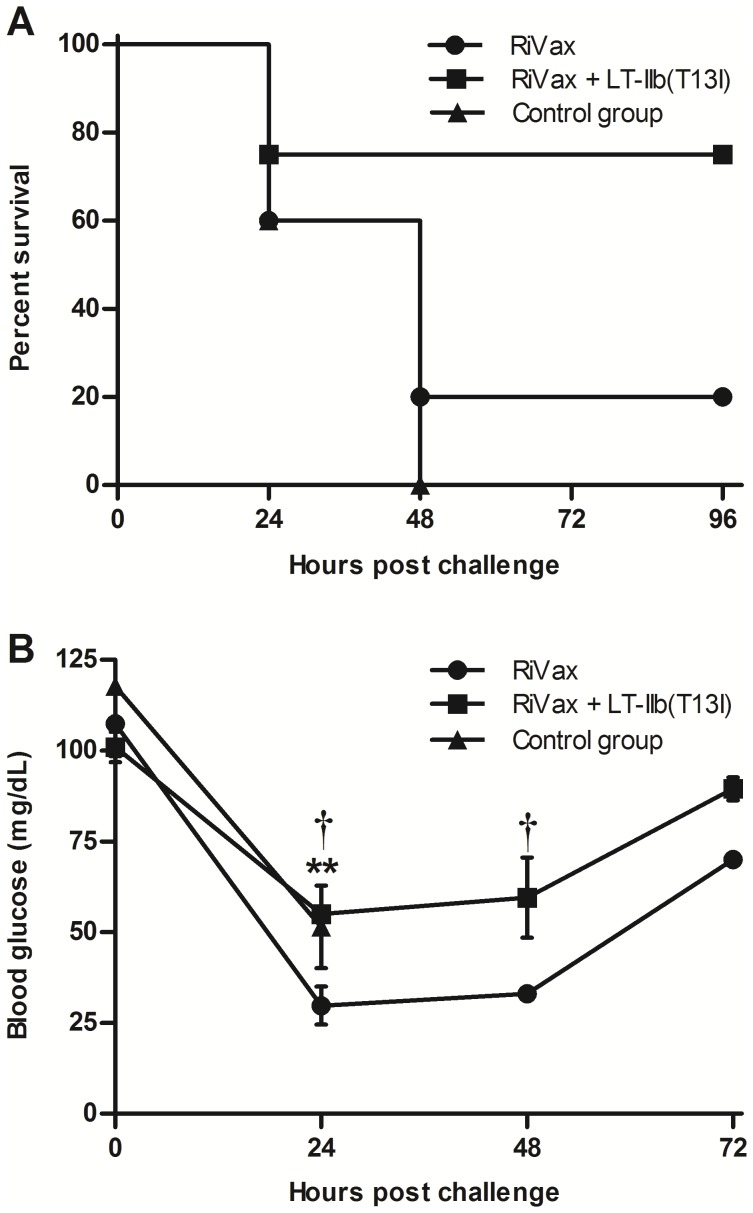
I.n. immunization of RiVax with LT-IIb(T13I) enhances protective immunity to ricin. Mice were immunized intranasally on days 0, 10, 20, and 34 with 0.5 µg of RiVax in the presence or absence of 1.0 µg of LT-IIb or LT-IIb(T13I) and challenged 14 days after the final immunization with 10 LD_50_ of ricin. (A) Survival of mice after i.p. challenge with ricin. Data were compared by the Logrank test. (B) Blood glucose levels of immunized mice during ricin challenge. Data are presented as the arithmetic mean with error bars denoting one S.E.M. (n = 4 or 5) Key: **, p<0.01 compared to RiVax; †, p<0.01 compared to 0 h. Data were compared using an unpaired Students t-test.

To further explore the potential of LT-IIb(T13I) to provide a dose-sparing effect when administered intranasally, i.n. immunization studies were repeated using a 10-fold lower dose of RiVax (0.5 µg). Not surprisingly, mice that received the low dose vaccine by the i.n. route did not develop Ag-specific Ab as rapidly as mice that had been immunized by the i.d. route. A third boost by the i.n. route was required to significantly raise anti-RTA titers; however, neutralization activity was not evident in any of the sera from immunized mice (data not shown). Two weeks after the third boost, immunized mice were challenged with 10 LD_50_ of ricin by i.p. injection and monitored for survival. All of the control mice and 80% of the mice that received 0.5 µg of RiVax by the i.n. route succumbed to intoxication by 48 h post challenge (**Fig. 7A**). Mice that received RiVax in combination with LT-IIb(T13I) exhibited enhanced protection (75% survival). Although the mice that received RiVax with LT-IIb(T13I) by the i.n route exhibited greater protection against ricin challenge than the other cohorts in the study, blood glucose levels in these mice also declined dramatically following ricin challenge, which demonstrated that the mice were only partially immune to the metabolic effects of the toxin (**Fig. 7B**). Taken together, these data indicated that combining RiVax with LT-IIb(T13I) enhanced protection when administered by the i.n. route, although the level of protection that is achieved is not as complete as in mice immunized with RiVax and LT-IIb(T13I) by the i.d. route.

## Discussion

In this study, we evaluated the capacity of LT-IIb and LT-IIb(T13I) to augment Ag-specific immune responses against RiVax, a candidate ricin toxin vaccine Ag. When co-administered via the i.d. route with RiVax, LT-IIb and LT-IIb(T13I) significantly enhanced anti-RiVax serum IgG levels (**Figs. 1** **and** **4**) in comparison to the levels in mice that received RiVax alone. Additionally, both adjuvants enhanced RiVax- specific IgG Ab in the lungs, thus indicating the likelihood of enhanced protection at this important mucosal surface. Given the lethality of ricin, the capacity of LT-IIb and LT-IIb(T13I) to enhance the levels of anti-RiVax Ab in the serum and in the lungs using an accelerated immunization regimen provides a strong advantage over other adjuvants that have been evaluated. Moreover, in comparison to the use of RiVax alone, LT-IIb and LT-IIb(T13I) also preferentially stimulated the production of ricin-neutralizing Ab, which was correlated with a strong augmentation in protective immunity to ricin challenge (**Figs. 2** **and** **3**). The fact that LT-IIb(T13I), when employed as an i.d. adjuvant, enhanced survival of mice lethally challenged with ricin justifies the further development of this adjuvant for use with RiVax and other subunit vaccines aimed at biological threat agents such as anthrax.

Co-administration of RiVax with detoxified LT-IIb(T13I) by either the i.d. route or i.n. route enhanced protection to a lethal systemic ricin challenge, in comparison to the level of protection observed in mice that had received only RiVax (**Figs. 3** **and** **7**). Although immunization by either route induced similar levels of Ag-specific IgG Ab, it is intriguing that immunization with RiVax and LT-IIb(T13I) by the i.n. route was less effective than the i.d. route at stimulating protection against ricin intoxication. This difference in protective capacity elicited by administration at the two sites may be a result of functional differences in the anatomical and immunological organization of the cells and tissues at those sites. For example, the skin harbors unique subsets of dendritic cells and large numbers of macrophages [32]. When RiVax and LT-IIb(T13I) are administered by the i.d. route, either of those types of cells may promote increased or altered Ag processing/presentation of protective epitopes.

As noted above, the cellular mechanism(s) by which LT-IIb(T13I) augments ricin-neutralizing Ab when employed as an adjuvant is unclear. LT-IIb(T13I) may preferentially promote the production of Ab to one or more neutralizing epitopes on RiVax. Additionally, LT-IIb(T13I), by virtue of its ability to bind to Ag presenting cells, may accelerate Ag uptake, processing, or presentation. Or LT-IIb(T13I) may accelerate antibody affinity maturation in B cells. Any one, or a combination of those mechanisms evoked by LT-IIb(T13I), could augment the production of Ab to neutralizing epitopes that would be ignored or minimally processed by immune cells in mice that did not receive the adjuvant. It will be intriguing to determine which of those mechanisms are modulated by LT-IIb(T13I).

Additionally, molecular mechanisms by which LT-IIb and LT-IIb(T13I) enhance production of cytokines and/or chemokines that favor production and affinity maturation of Ab have also not been well-described. Interleukin-6 (IL-6), a potent B cell differentiation factor that is produced by many cell types, drives B cell maturation and stimulates Ab production [39]. Indeed, LT-IIb and LT-IIb(T13I) (T.D.C. and C.J.G., unpublished) induces robust production of IL-6 in several cell populations including mononuclear cells and lymphocytes [28], [30], [40]. Whether LT-IIb(T13I)-induced cytokines/chemokines influence immune functions locally in the skin or within regional draining lymph nodes to enhance Ag-specific immune responses remains to be determined.

Although alum has a long history of success as an i.m. adjuvant, this salt preparation often induces long-lasting granulomas at the injection site and elicits local allergic reactions [41], [42]. In comparison to LT-IIb and Imject®, LT-IIb(T13I) exhibited a decreased propensity to promote inflammation at the site of immunization (**Fig. 5**). In fact, the skin sections from LT-IIb(T13I) recipient mice were largely indistinguishable from skin sections obtained from mice that had received only RiVax just one week after immunization. Additionally, the reduced injection site indurations induced by LT-IIb(T13I) resolved much quicker when compared to the indurations induced by Imject®. While the mechanisms by which LT-IIb(T13I) augments Ag-specific immune responses in the skin have not been elucidated, it is feasible that LT-IIb(T13I), by its decreased affinity for ganglioside receptors compared to LT-IIb, fails to induce high levels of inflammatory cytokines at the site of immunization. Experiments addressing these questions are ongoing.

In summary, this study demonstrated that LT-IIb and the detoxified mutant LT-IIb(T13I) are potent i.d. adjuvants when co-administered with RiVax, a prospective vaccine candidate against ricin. LT-IIb and LT-IIb(T13I) not only enhanced the production of anti-RiVax Ab when administered by the i.d. route, but also increased the levels of ricin-neutralizing Ab in the serum. When administered in the skin, LT-IIb(T13I) was much less inflammatory than LT-IIb. Importantly, LT-IIb(T13I) elevated the ability of RiVax to induce protective immunity to a lethal challenge of ricin. Taken together, these data support the potential use of LT-IIb(T13I) as an effective next-generation i.d. adjuvant. Future studies will evaluate the effectiveness and safety of i.d. administration of LT-IIb(T13I) with RiVax in additional animal models and ultimately, in humans. It will also be critical to determine if LT-IIb(T13I) augments production of neutralizing Ab and enhances protection against other bioterrorist agents and public health pathogens (e.g., anthrax, botulinum neurotoxin, plague, HIV, etc.).
